# Prevalence, awareness, and patterns of non-steroidal anti-inflammatory drug use among health science students in Palestine: a cross-sectional study

**DOI:** 10.1038/s41598-023-47279-2

**Published:** 2023-11-13

**Authors:** Aseel I. Aboalrob, Falasteen M. Eid, Saba M. Esa, Amer A. Koni, Samah W. Al-Jabi, Sa’ed H. Zyoud

**Affiliations:** 1 Department of Clinical and Community Pharmacy, College of Medicine and Health Sciences, An-Najah National University, Nablus, 44839 Palestine; 2 Division of Clinical Pharmacy, Department of Hematology and Oncology, An-Najah National University Hospital, Nablus, 44839 Palestine; 3 Poison Control and Drug Information Center (PCDIC), College of Medicine and Health Sciences, An-Najah National University, Nablus, 44839 Palestine; 4 Clinical Research Center, An-Najah National University Hospital, Nablus, 44839 Palestine

**Keywords:** Health occupations, Medical research

## Abstract

Despite the widespread use of nonsteroidal anti-inflammatory drugs (NSAIDs), many people lack sufficient awareness regarding their side effects and proper usage. Consequently, this study aimed to assess the knowledge, behavior, and usage patterns of NSAIDs among students enrolled in Palestinian health colleges. The study was conducted in December 2020 using a cross-sectional design, and a convenience sampling method was employed to enroll a total of 206 students. The questionnaire comprised seven sections, each containing approximately 5 to 10 closed-ended questions. Data were analyzed using version 21 of the Statistical Package for the Social Sciences (IBM-SPSS Statistics 21). The percentage of respondents who admitted that NSAIDs were used several times in the year was 35.9%. More than half of the students believed that NSAIDs were generally safe. Seventy-two percent of the students were aware that more than one type of NSAID at the same time increased the side effects. The median knowledge score of NSAID side effects was 9 [6.25–11] out of 13. The knowledge score increased significantly with age (*p* < 0.001), higher academic year (*p* < 0.001), and pharmacy and medicine specialties (*p* = 0.002). The college students surveyed in this study possess a general awareness of NSAIDs. Nonetheless, there remains a necessity to enhance their behavior and practices concerning the utilization of NSAIDs through the implementation of programmed educational strategies.

## Introduction

Nonsteroidal anti-inflammatory drugs (NSAIDs) are presently regarded as one of the most widely used medications due to their antipyretic, anti-inflammatory, and analgesic properties. These drugs are conveniently available as over-the-counter (OTC) medications^1,2^. Epidemiological research indicates that there is a significant usage of both prescribed and OTC analgesics among individuals^3^. Many young adults frequently resort to NSAIDs and paracetamol for self-therapy to alleviate diverse ailments, such as pain, fever, and inflammatory disorders. However, there is a concerning trend wherein NSAIDs are utilized in numerous instances where they are contraindicated, owing to the rising usage of analgesics^4^.

Gastrointestinal (GI) complications in conjunction with cardiovascular, hepatic, renal, and other toxicities will continue to be reported^2^. Furthermore, several studies have shown that patients lack sufficient information and awareness about the risks that may occur due to therapy itself and how good monitoring should be applied concurrently with treatment^5,6^. According to one study, over one-third of orthopedic patients reported insufficient information regarding side effects. Additionally, more than 90% of the participants agreed that pharmacists and physicians should actively educate patients about ADRs^6^. The findings of a systematic literature search across various databases revealed a significantly low level of awareness regarding NSAIDs^7^.

Recently, some studies have focused on consumption and awareness among students^4,8^. While there are no recent data available on the most commonly used over-the-counter medications in Palestine, it has been reported that NSAIDs are frequently used in various populations^9^. Ibuprofen and diclofenac were the most commonly used NSAIDs, mainly purchased without a prescription. On the other hand, the study found that selective cyclooxygenase-2 (COX-2) inhibitors were the most commonly prescribed NSAIDs^10^. A previous study revealed a high prevalence (79.3%) of NSAID usage among Palestinian students. Importantly, the study also reported a lack of awareness regarding the side effects of NSAIDs^11^. Furthermore, it documented a significant deficiency in the information provided by local NSAID package inserts, including details about side effects, warnings, and contraindications^12^.

No studies have been conducted in Palestine to assess medical students' awareness and attitudes toward using NSAIDs. Therefore, we surveyed to investigate the knowledge, consumption, and awareness of NSAIDs among Palestinian health students and to determine the factors that influence this knowledge. This study aims to document and identify how Palestinian university students from health fields use and misuse NSAIDs, evaluate their understanding of these medications, and determine their awareness of NSAIDs. Given that these students will play a crucial role in the future as healthcare providers in our society, it is important to guide them in promoting the correct use of medications. Additionally, the findings from this study will aid in developing and implementing educational strategies focused on improving medical students' knowledge about appropriate usage and potential side effects.

## Methods

### Study design

The study was cross-sectional and was conducted in December 2020. A questionnaire with closed-ended questions was used for data collection through face-to-face interviews.

### Study setting and study population

This survey was carried out on health college students at An-Najah National University (NNU), a nongovernmental public university in Nablus, which is a city in the north of the West Bank, Palestine. The university has a student body of 25,000, and its Faculty of Medicine and Health Sciences consists of six departments: Biomedical Sciences, Medicine, Dentistry and Surgery, Applied and Allied Medical Sciences, Pharmacy, and Nursing and Midwifery.

### Sample size and sample technique

In the current study, the total number of university students in medical colleges was approximately 5000. The sample size was determined to be 357 using an electronic sample size calculator called Raosoft. However, we could not reach this target due to the implementation of COVID-19 safety measures. Consequently, a convenience sample of 206 students from the health college at NNU was selected, comprising 60 males and 146 females.

### Inclusion and exclusion criteria

The study enrolled students studying at NNU who were enrolled in medical colleges. However, participants from other universities, those in the concession period (an additional year of training after graduation for medical students), and individuals who had never taken any NSAIDs throughout their lives were excluded.

### Data collection instrument

Data were collected by three PharmD students who were in the last year of their studies and were extensively trained to follow the data collection protocol. The questionnaire comprised a comprehensive table listing all the commercial names of NSAIDs and paracetamol currently accessible in Palestine. Participants were needed to select at least one medication they had previously consumed. The questionnaire was divided into seven sections to facilitate organization, each consisting of multiple questions derived from prior research studies^4,6,13^ (Additional file 1). The first section had questions about sociodemographic characteristics, including age, sex, residency, living status, social status, specialization, insurance, income, and academic year. The second section asked about the frequency and indications of using NSAIDs and paracetamol. The third section consisted of questions that measured the patterns of NSAID consumption; for example, participants were asked about the dosage forms they usually used (pill, injection, solution, and suppository), ways to obtain the drug (from the doctor, pharmacy, remnant drugs, university clinic, family, and friends) and substances that were taken concomitantly with NSAIDs (coffee, herbs, and other substances). The fourth section consisted of seven closed-ended questions that measured participants’ perception of drug safety and evaluated their opinion about E-marketing and the availability of NSAIDs in places other than pharmacies. The fifth section investigated the patterns of NSAID use (continuously or intermittently), attitudes about pain relief, sources of information, and increasing the dose of NSAIDs. The sixth section evaluated participants' knowledge about side effects and the impacts of certain clinical conditions on these side effects. This part contained 13 questions to be answered using ‘true/false/I do not know’ options. Participants received one point for the correct answer and zero points for the other options. The resulting knowledge scale ranged between 0 and 13, with a higher score indicating good knowledge. The last section investigated participants' attitudes toward receiving information about adverse drug reactions to NSAIDs, where 17 items were used and responded using strongly agree, agree, not sure, disagree or strongly disagree options.

### Validity/Pilot study

The questionnaire's content was evaluated by three expert researchers from clinical pharmacy academics who have practical experience in community pharmacy and are qualified in this type of research. They assessed medical terms, questionnaire organization, completeness, and suitability. Cronbach's alpha of the knowledge questions was 0.755. After modifying the content and design of the questionnaire, it was tested on ten health college students who were not involved in the final analysis. The pilot and final questionnaires were conducted in Arabic, and each participant took approximately 10 min to complete all questions through face-to-face interviews.

### Ethical issues

Official approval was obtained from the *Institutional Review Board (IRB) of An-Najah National University* before data collection began (IRB approval number: *Phd. Sep./2020/2*). We provided the participants with a detailed explanation of the study objectives and confirmed that the data would not be used for purposes other than scientific research. Then, informed verbal consent was obtained while maintaining the confidentiality of the data. Furthermore, we provided the respondents with a detailed explanation of the questionnaire and encouraged them to provide accurate and correct information.

### Statistical analysis

Data were analyzed using version 21 of the Statistical Package for the Social Sciences (IBM-SPSS Statistics 21). The results were mainly described as frequencies and percentages. The normality of all scores was checked using the Kolmogorov‒Smirnov test, which showed that the data were not normally distributed. Therefore, the median and percentiles were adopted to express the variables, and the Kruskal‒Wallis U and Mann‒Whitney tests were used to check the differences in knowledge scores between categories of each variable as appropriate. A statistically significant p-value was assumed to be less than 0.05.

## Results

### Demographic data

The questionnaire comprised 206 medical college students with an average age of 20.81 ± 1.73 years in various fields. In this study, the majority of the respondents (70.4%) were females, 53.4% lived in villages, 44.2% lived in camps, and 95.5% were single. Additional demographic data are shown in Table 1.

**Table 1:** Demographic data for those using NSAIDs.

Characteristics	Frequency (%)
Did you use NSAIDs before?	
Yes	206 (95.4%)
NO	10 (4.6%)
Age categories (years)	
≤ 19	42 (20.4)
20–21	105 (51.0)
≥ 21	59 (28.6)
Gender	
Male	61 (29.6)
Female	145 (70.4)
Residency	
Camp	91 (44.2)
Village	110 (53.4)
City	5 (2.4)
Living status	
Alone	17 (8.3)
With my family	183 (88.8)
Others	6 (2.9)
Social status	
Single	197 (95.6)
Married	9 (4.4)
Specialization	
Medicine	43 (20.9)
Pharmacy*	52 (25.2)
Laboratory science	12 (5.8)
Nursing/midwifery	43 (20.9)
Pharm.D.*	23 (11.2)
Speech and hearing	4 (1.9)
Optics	18 (8.7)
Physiotherapy	11 (5.3)
Insurance	
I don’t have insurance	66 (32)
Private insurance	34 (16.5)
Governmental insurance	106 (51.5)
Income	
Less than 2000 NIS	13 (6.3)
2000–5000 NIS	106 (51.5)
5000–10,000 NIS	61 (29.6)
More than 10,000 NIS	26 (12.6)
Education level	
1st year	17 (8.3)
2nd year	29 (14.1)
3rd year	44 (21.4)
4th year	65 (31.6)
5th year	24 (11.7)
6th year	27 (13.1)

*Pharm.D. : Doctors of Pharmacy (Pharm.D. program focuses on the clinical aspects of medications. It is a 6-year certificate granted to graduates who completed one additional year of clinical training in the hospital departments. However, pharmacy graduates study 5 years with more focus on the pharmaceutical aspects of medications); NIS: New Israeli Shekel.

### Indications for taking NSAIDs and paracetamol

The responses to the questions regarding the indications for taking NSAIDs are shown in Table 2. In response to the question ‘the frequency of NSAID use’, 35.9% admitted that NSAIDs were used several times in the year, while 23.8%, 21.4%, and 16% of the respondents took NSAIDs once a year, once a month, and several times a month, respectively. The reasons for NSAID use by students were as follows: toothache (50.5%), fever (41.8%), menstrual pain (53.8%), muscle pain (34%), common cold (38.3%) and posttraumatic stress disorder (27.2%). It was also noted that under certain conditions, a minority of students took NSAIDs in combination with paracetamol, such as the common cold (12.1%), headaches (8.7%), and fever (8.3%) (Table 2).

**Table 2:** Indications for taking NSAIDs and paracetamol.

Questions	Frequency (%)
Cold cases	
NSAIDs only	54 (26.2)
Paracetamol only	93 (45.1)
Both	25 (12.1)
Neither	34 (16.5)
Toothache	
NSAIDs only	88 (42.7)
Paracetamol only	66 (32.0)
Both	16 (7.8)
Neither	36 (17.5)
Fever	
NSAIDs only	69 (33.5)
Paracetamol only	86 (41.7)
Both	17 (8.3)
Neither	34 (16.5)
Stomach pain or heartburn	
NSAIDs only	33 (16.0)
Paracetamol only	36 (17.5)
Both	3 (1.5)
Neither	134 (65.0)
Muscle pain	
NSAIDs only	64 (31.1)
Paracetamol only	42 (20.4)
Both	6 (2.9)
Neither	94 (45.6)
Stress	
NSAIDs only	17 (8.3)
Paracetamol only	69 (33.5)
Both	5 (2.4)
Neither	115 (55.8)
Headaches	
NSAIDs only	20 (9.7)
Paracetamol only	145 (70.4)
Both	18 (8.7)
Neither	23 (11.2)
Asthma	
NSAIDs only	22 (10.7)
Paracetamol only	24 (11.7)
Both	1 (0.5)
Neither	159 (77.2)
Menstrual pain *for female only*	
NSAIDs only	78 (53.8)
Paracetamol only	34 (23.4)
Both	5 (3.4)
Neither	28 (19.3)

### Consumption patterns of NSAIDs

The most common dosage form used by the respondents was pills (96.6%). Furthermore, 80.6% of students received NSAIDs from the pharmacy, while others received NSAIDs from doctors, remnants of previous medications, university clinics, friends, or family medications. Although NSAIDs were sometimes taken with other substances, most students (81.1%) took them alone (Table 3).

**Table 3:** Student consumption pattern of NSAIDs.

Question	Frequency
Are you used to taking NSAIDs in pill form?	
Yes	199 (96.6)
No	7 (3.4)
Are you used to using NSAIDs in injection form?	
Yes	24 (11.7)
No	182 (88.3)
Do you get NSAIDs from the pharmacy?	
Yes	166 (80.6)
No	40 (19.4)
Do you get NSAIDs from the doctor?	
Yes	37 (18.0)
No	169 (82)
Do you get NSAIDs from the remnants of previous medications?	
Yes	12 (5.8)
No	194 (94.2)
Do you get NSAIDs from the university clinic?	
Yes	10 (4.9)
No	196 (95.1)
Do you get NSAIDs from family medications?	
Yes	36 (17.5)
No	170 (82.5)
Do you get NSAIDs from your friends?	
Yes	5 (2.4)
No	201 (97.6)
Do you take NSAIDs alone?	
Yes	167 (81.1)
No	39 (18.9)
Are you taking NSAIDs in conjunction with coffee?	
Yes	26 (12.6)
No	180 (87.4)

### View of respondents on the safety and availability of NSAIDs

Most participants (55.3%) thought NSAIDs were generally safe, and most (74.8%) believed they knew about their side effects. Approximately seventy-seven percent knew that NSAIDs could be purchased without a prescription.

Regarding the marketing of NSAIDs through social media and buying them from different places, such as supermarkets, most of the participants thought it was not appropriate (87.4% and 91.7%, respectively). A total of 12.6% believed it appropriate to market anti-inflammatory drugs via social media (Table 4).

**Table 4:** View of respondents on the safety and availability of NSAIDs.

Questions	N (%)
Do you think it is generally safe to use NSAIDs?	
Yes	114 (55.3)
No	92 (44.7)
Do you know the side effects of using NSAIDs?	
Yes	154 (74.8)
No	52 (25.2)
Do you think NSAIDs can be purchased without a prescription?	
Yes	159 (77.2)
No	47 (22.8)
Do you think that easy access to NSAIDs ‘as happens these days’ is suitable?	
Yes	102 (49.5)
No	104 (50.5)
Do you think it is appropriate to buy NSAIDs from different places such as the supermarket?	
Yes	17 (8.3)
No	189 (91.7)
Do you think NSAIDs are being abused at present?	
Yes	178 (86.4)
No	28 (13.6)
Do you think it is appropriate to market anti-inflammatory drugs via social media?	
Yes	26 (12.6)
No	180 (87.4)

### Student behavior patterns when using NSAIDs

A total of 15.1% of medical college students emphasized their continuous use of NSAIDs, and a high percentage (99%) reported their use as needed. Approximately 59.2% of participants relied on the pharmacist as the first source of information when buying NSAIDs without a prescription, 56.3% asked the doctor, and 21.4% relied on drug brochures. Only 13.1% asked their families, and 3.3% obtained drug information from social networks (Table 5).

**Table 5:** Student behavior patterns when taking NSAIDs.

Questions	N (%)
When using NSAIDs, do you use them continuously or intermittently?	
Continuously	31 (15.1)
Intermittently	175 (84.9)
How to use painkillers?	
Only when needed	204 (99)
Chronic use	2 (1)
If the pain does not go away, do you ask the doctor for counseling?	
Yes	85 (41.3)
No	121 (58.7)
If the pain does not disappear, do you ask for pharmacist counseling?	
Yes	24 (11.7)
No	182 (87.5)
If the pain does not subside, do you take a second dose?	
Yes	86 (41.7)
No	120 (58.2)
If the pain does not disappear, do you use another painkiller?	
Yes	32 (15.5)
No	174 (84.4)
When you buy NSAIDs without a prescription, is the pharmacist your first source of information?	
Yes	122 (59.2)
No	84 (40.7)
When you buy NSAIDs without a prescription, is the doctor your first source of information?	
Yes	116 (56.3)
No	90 (43.6)
When you buy NSAIDs without a prescription, are books and research your first source of information?	
Yes	39 (18.9)
No	167 (81.0)
When you buy NSAIDs without a prescription, are social media your first source of information?	
Yes	7 (3.3)
No	199 (96.7)
When you buy NSAIDs without prescription, is the family your first source of information?	
Yes	27 (13.1)
No	179 (86.9)
When you buy NSAIDs without a prescription, are your friends your first source of information?	
Yes	2 (1)
No	204 (99.0)
When you buy NSAIDs without prescription, is the leaflet your first source of information?	
Yes	44 (21.4)
No	162 (78.6)
Do you think that increasing the dose of painkillers may have side effects?	
Yes	189 (91.7)
No	8 (3.8)

### Knowledge of health college students about the side effects of NSAIDs

Most of the participants were aware of the side effects of NSAIDs on the kidney (92.2%), cardiovascular system (80.6%), and peptic ulcers (82.5%). Only 21.4% correctly identified that NSAIDs do not affect the growth of cancer cells. Additionally, 71.8% of the respondents knew that taking more than one type of NSAID at the same time increases their side effects, and 70.4% were aware of the increased risk of side effects in elderly users (70.4%). Other results related to patient knowledge are shown in Table 6.

**Table 6:** Knowledge of college students about the side effects.

Question	Answer	Correct answer N (%)	Incorrect answer N (%)	I don’t know N (%)
The incorrect use of NSAIDs could be harmful to the kidneys	T	190 (92.2)	5 (2.4)	11 (5.3)
The incorrect use of NSAIDs Could be harmful to the cardiovascular system	T	166 (80.6)	9 (4.4)	31 (15)
The incorrect use of NSAIDs makes cancer cells grow faster	F	44 (21.4)	60 (29.1)	102 (49.5)
The incorrect use of NSAIDs causes peptic ulcers	T	170 (82.5)	6 (2.9)	30 (14.6)
The incorrect use of NSAIDs causes gastrointestinal bleeding	T	127 (61.7)	19 (9.2)	60 (29.1)
The incorrect use of NSAIDs is harmful to the respiratory system	T	93 (45.1)	37 (18)	76 (36.9)
NSAIDs Can be used in one of the stages of pregnancy	T	55 (26.7)	111 (53.9)	40 (19.4)
The side effects of NSAIDs increases in the elderly	T	145 (70.4)	13 (6.3)	48(23.3)
Taking a high dose of NSAIDs increases their side effects	T	173 (84)	12 (5.8)	21 (10.2)
Taking NSAIDs chronically increases their side effects	T	173 (84)	11 (5.3)	22 (10.7)
Taking more than one type of NSAID at the same time increases their side effects	T	148 (71.8)	18 (8.7)	40 (19.4)
The side effects of NSAIDs increase in patients with hypertension and heart disease	T	140 (68)	8 (3.9)	58 (28.2)
Smokers are more likely to have side effects from NSAIDs	T	92 (44.7)	16 (7.8)	98 (47.6)

*T* true, *F* false.

### Knowledge score by sociodemographic variables

The students' median [interquartile range] knowledge score was 9 [6.25–11]. Statistical analysis indicated a significant relationship between age (*p* < 0.001), specialty (*p* = 0.002), and academic year (*p* < 0.001) and the knowledge score. For example, pharmacy students had better knowledge scores than other health college students, followed by PharmD and medical students. Fifth- and sixth-year students had the highest knowledge scores (Table 7).

**Table 7:** Knowledge score by sociodemographic variables.

Characteristic	Median (Q1-Q3)	P value	Test statistic*	Post hoc analysis; significant pairs
Age categories (years)		(< 0.001)^b^	32.362	a-b; b-c; a-c
≤ 19 (a)	6 (4–9)			
20–21 (b)	9 (7–11)			
≥ 21 (c)	10 (8–11)			
Gender		(0.214)^a^	-1.243	none
Male	8 (6.25–10)			
Female	9 (6.5–11)			
Residency		(0.534)^b^	1.254	none
Camp	9(5–10)			
Village	9 (7–11)			
City	8 (6–11)			
Living status		(0.423)^b^	1.720	none
Alone	8 (7–10)			
With my family	9 (6–11)			
Others	10 (8.5–11)			
Social status		(0.061)^a^	-1.871	none
Single/divorced	9 (6–11)			
Married	10 (8.5–11.5)			
Specialization		(0.002)^b^	22.186	a-d, a-f, a-h, b-d, b-f, b-g, b-h, e-d, e–f, e–g, e–h
Medicine (a)	9 (7–11)			
Pharmacy (b)	10 (8–11)			
Lab. Science (c)	8 (7–10)			
Nursing/midwifery (d)	8 (5–10)			
Pharm.D. (e)	9 (8–11)			
Speech and hearing (f)	6 (5.25–7.5)			
Optics (g)	7 (5–10)			
Physiotherapy (h)	6 (4–9)			
Insurance		(0.412)^b^	1.775	none
I don’t have insurance	9 (7–11)			
Private insurance	8 (6–10)			
Governmental insurance	9 (6–11)			
Income		(0.591)^b^	1.914	none
Less than 2000 NIS	8 (6–9.5)			
2000–5000 NIS	9 (6–11)			
5000–10,000 NIS	9 (7–11)			
More than 10,000 NIS	8.5 (7–11)			
Education level		(< 0.001)^b^	39.759	a-d, a-e, a-f, b-c, b-d, b-e, b-f, c-e, c-f
1st year (a)	7 (4–9)			
2nd year (b)	5 (4.5–8.5)			
3rd year (c)	9 (6.25–10)			
4th year (d)	9 (7–11)			
5th year (e)	10.5 (8–11)			
6th year (f)	10 (9–11)			

*The Mann–Whitney Z/Kruskal–Wallis χ^2^ score.

^a^The statistical significance of the differences was calculated using the Mann‒Whitney U test.

^b^The statistical significance of the differences was calculated using the Kruskal‒Wallis test.

### Attitudes toward receiving information about the side effects of NSAIDs

All participants agreed on the importance of having information about side effects, with 75.7% agreeing strongly. A total of 56.8% of students believed that informing them about side effects increased their anxiety. Additionally, 63.6% and 54.4% of the students strongly agreed that physicians and pharmacists have an important role in providing information to patients about side effects. Many of the students (80.6%) ensure that there is a need for a leaflet for all medicines, and 45.1% and 23.3% agree and strongly agree, respectively, that they will read it carefully when available. However, some students (16.5%) considered the leaflets to be an inaccurate source of information, and 30.6% rated the previous leaflets that had been read as difficult to understand (Table 8).

**Table 8:** Attitudes toward receiving information about the side effects of NSAIDs.

Question	N (%)
It is important to have an idea about medication side effects	
Strongly agree	156 (75.7)
Agree	50 (24.3)
I am not sure	0 (0.0)
Disagree	0 (0.0)
Strongly disagree	0 (0.0)
Informing you of the side effects of the medication may increase your level of anxiety	
Strongly agree	29 (14.1)
Agree	88 (42.7)
I am not sure	28 (13.6)
Disagree	51 (24.8)
Strongly disagree	10 (4.9)
The doctor should have a role in informing you about the side effects of the medicines	
Strongly agree	131 (63.6)
Agree	63 (30.6)
I am not sure	5 (2.4)
Disagree	6 (2.9)
Strongly disagree	1 (0.5)
The pharmacist has a direct role to play in providing information about the side effects of the medicine	
Strongly agree	112 (54.4)
Agree	63 (30.6)
I am not sure	22 (10.7)
Disagree	5 (2.4)
Strongly disagree	4 (1.9)
Having information about the side effects of medication helps the patient to inform the doctor about the abnormal symptoms that may appear	
Strongly agree	120 (58.3)
Agree	71 (34.5)
I am not sure	8 (3.9)
Disagree	3 (1.5)
Strongly disagree	4 (1.9)
Having information about the side effects of your medicine may cause you to stop using the medicine	
Strongly agree	33 (16)
Agree	68 (33)
I am not sure	49 (23.8)
Disagree	47 (22.8)
Strongly disagree	9 (4.4)
Receiving information from the drug leaflet may encourage noncompliance with the medicine	
Strongly agree	17 (8.3)
Agree	28 (13.6)
I am not sure	57 (27.7)
Disagree	83 (40.3)
Strongly disagree	21 (10.2)
The drug leaflet is a source of information through which it is possible to monitor the side effects of a medicine and inform the doctor about them	
Strongly agree	52 (25.2)
Agree	114 (55.3)
I am not sure	23 (11.2)
Disagree	15 (7.3)
Strongly disagree	2 (1)
Receiving information from the drug leaflet if a medicine is used for the first time is necessary	
Strongly agree	81 (39.3)
Agree	82 (39.8)
I am not sure	22 (10.7)
Disagree	19 (9.2)
Strongly disagree	2 (1)
The leaflet information is an inaccurate source of medication information for you	
Strongly agree	8 (3.9)
Agree	26 (12.6)
I am not sure	31 (15)
Disagree	104 (50.5)
Strongly disagree	37 (18)

## Discussion

The side effects associated with NSAIDs are deemed significant and occur alarmingly. These agents are widely known to cause gastrointestinal toxicity, including peptic ulcers. NSAID users should be aware of this side effect, especially patients who have a prior history of peptic ulcers^14^. Furthermore, they can trigger hypersensitivity reactions, manifesting as rhinosinusitis and asthma. In severe cases, these reactions can escalate to anaphylaxis. Additionally, NSAIDs, particularly selective COX-2 inhibitors, can elevate the risk of blood thrombosis through their mechanism, leading to numerous complications that may result in myocardial infarction^4^. In the past, NSAIDs' gastrointestinal (GI) side effects posed a significant limitation to their usage. NSAIDs have been associated with various cardiovascular and renal conditions, including sodium and water retention in individuals with hypertension and acute and chronic kidney disease^15^. Although these agents can cause various side effects when used regularly and in patients with comorbidities, their effectiveness and the advertising efforts of pharmaceutical companies contribute to their high popularity among the public^8^.

In addition, little data on the level of knowledge, attitudes, and characteristics of the population that uses them, especially younger individuals, are available^5^. Therefore, in this study, our objective was to evaluate the knowledge of medical college students who are expected to be the most educated about NSAIDs and to assess their behavior around their use and misuse, which is essential to developing and targeting a suitable educational intervention. In this context, it is worth noting that pharmacy and PharmD students and medicine students achieved the highest knowledge scores. On the other hand, speech and hearing, as well as physiotherapy students, obtained the lowest scores. These findings align with the Ontario Physiotherapist Survey, which revealed that a mere 2.8% of physiotherapists provided correct recommendations, indicating a lack of sufficient knowledge among the majority^16^. Most physiotherapists in Pakistan have poor knowledge of NSAID prescriptions and recommend, advise, and prescribe NSAIDs to their patients, leading to adverse reactions, misuse, and poor knowledge^17^. In Palestine, the prescription of NSAIDs should be monitored, and a strict policy should be followed, as healthcare workers, such as physiotherapists and nurses, sometimes prescribe these medications.

In the present analysis, the percentage of students who had previously used NSAIDs was as high as 95.4%. A previous study conducted among university students reported that 98.8% emphasized their active utilization of OTC medications, including NSAIDs and paracetamol^4^. Likewise, a study indicated that 94% of Irish student-athletes had previously utilized NSAIDs^8^. However, the prevalence of NSAID use among Jordanian university students showed that 31.3% used NSAIDs, while 46.5% did not, and 22.2% did not know if they used NSAIDs or not^18^. Moreover, the utilization rate of NSAIDs among Palestinian athletes was recorded at 79.3%^11^, while it stood at 77% among Polish medical students^19^ and 68% among Iranian university students^20^. Our research observed that the frequency of NSAID usage, either once weekly or several times a week, was 3%. Compared to the Jordanian population, the majority of users (81%) took NSAIDs once a month or less, while 10.5% used them weekly, 4.6% used them two to three times a week, and 3.7% used them daily^21^.

A majority of our participants utilized pill dosage forms for NSAIDs, while other forms were less commonly used, aligning with a prior report^19^. Additionally, our study indicated that approximately half of the respondents regarded NSAIDs as safe medications^19^, and another study revealed that 52% of the students held the same perception^4^. These findings closely resemble our findings. A previous study conducted among students in various fields found that nonsteroidal anti-inflammatory drugs (NSAIDs) were used in incorrect clinical conditions. For example, 1.2% of the participants used NSAIDs during asthma attacks, 2.4% for vomiting, 3.6% for malaise and depression, 14.0% in winter and fall as a preventive measure against infections, 2.0% for heartburn, and 16.0% for food poisoning. The study results also indicated that 36.0% of the students consistently read the information provided in the leaflet, while only 4.0% read it when using a new medication^4^. Importantly, 13.9% of Irish college athletes exceeded the recommended daily dose, particularly when using NSAIDs as over-the-counter drugs. Furthermore, 6.6% and 12.5% of the participants used NSAIDs for more than 14 and 10 days, respectively^8^. Additionally, previous research revealed that more than 50% of the participants were unaware of the potential side effects^8^. A study carried out in a rheumatology clinic hospital in Malaysia showed that half of the patients who attended this clinic were unaware of the side effects, and most educated patients were self-educated^22^. Approximately 77% of the participants in the current survey thought that NSAIDs could be bought without a prescription, which was higher than that in another study (40%)^19^.

In our study, the knowledge score was 9, accounting for 69.2% of the correct answers. This score can be regarded as low, particularly considering that health college students actively promote public awareness of medical matters. It is worth noting that pharmacy students obtained higher knowledge scores than other students, likely due to the curriculum they follow at university, which emphasizes this type of information. Previous studies have concluded that students' knowledge levels are insufficient and require improvement^4,8,11,19^.

Regarding attitudes toward receiving information about NSAID side effects, most participants expressed the significance of knowing NSAIDs. This aligns with a study conducted in patients in Thailand that aimed to assess attitudes toward receiving information about NSAIDs. In that study, a large percentage of respondents (98.1%) agreed that they should be aware of the potential side effects, and a significant number (96.8%) indicated a preference for receiving information about their initial prescription through leaflets^5^. Moreover, a significant proportion acknowledged the crucial role of doctors and pharmacists in delivering information regarding side effects. Additionally, over 70% recognized the importance of obtaining information from the drug leaflet, particularly when initiating drug usage. Nevertheless, some individuals encountered difficulty comprehending the information provided within the leaflet. Consequently, while the presence of a leaflet holds importance, it cannot substitute the role of doctors and pharmacists in furnishing crucial details on common side effects, as well as guidance on monitoring and prevention. Specifically, the leaflet encompasses all potential medication side effects, including rare occurrences, which may heighten patient anxiety and lead to nonadherence to the prescribed treatment. The percentage of female students at NNU is 66%, which is almost the same as our sample, 70.4%^23^; however, this can affect our results because females may be more familiar with drugs in general, and due to the presence of menstrual pain in females, they may be more likely to use NSAIDs than males, which will affect the result of the question related to the frequency of NSAID use yearly among students.

### Strengths and limitations

This study represents the first investigation conducted in Palestine to examine the awareness and usage patterns of NSAIDs among healthy college students. However, there are several limitations associated with the present study. First, a cross-sectional design was employed, which does not account for changes in student knowledge and attitudes over time. Second, the generalizability of the study findings is limited, as it focused solely on medical health college students at NNU, it did not allocate the sample size proportionally across the different departments, and there was a low frequency of NSAID use in this population. Third, the distribution of students interviewed across different educational levels was uneven. Moreover, despite achieving a high response rate, the sample size in this study was relatively small, and the prevalence of NSAID use among this population was not 100%. Although the content and face validity of the currently used tool were determined, it is not accompanied by the I-content validity index (I-CVI). Nonetheless, this study serves as an initial step in evaluating NSAID consumption and behaviors related to their use and misuse among university students in the health field. Future studies should aim to establish correlations between the level of awareness and patterns of NSAID use and sociodemographic and educational factors.

## Conclusions

Palestinian health college students at NNU generally possess a good understanding of NSAIDs. However, there remains a need to enhance their knowledge and behavior regarding the use of NSAIDs. This can be accomplished by implementing tailored educational strategies designed for university students. Moreover, the highest level of knowledge was observed among pharmacy students and those in advanced academic years. It is crucial to note that many patients acquire NSAIDs from pharmacies, underscoring the pivotal role of pharmacists in delivering accurate, specific, and understandable information to patients. This finding emphasizes the importance of pharmacists being highly qualified and well informed. Despite the widespread use of NSAIDs among medical college students, increasing their awareness regarding the appropriate utilization of these medications considering their significance within our society is imperative. We recommend the distribution of educational brochures related to NSAIDs in hospitals and medical colleges at the university, along with organizing lectures on this subject. These educational strategies should primarily focus on the appropriate use of NSAIDs through a prescription from physicians or pharmacists (as over-the-counter) and include information about the risk of using self-medication without appropriate consultation.

### Supplementary Information

Supplementary Information.

## Data Availability

The data sets supporting the results of the current research are available from the corresponding authors upon request.

## Abbreviations

NSAIDs: Nonsteroidal anti-inflammatory drugs
NNU: An-Najah National University
SPSS: The Statistical Package for the Social Sciences
OTC: Over-the-counter
GP: General practitioner
COX-2: Cyclooxygenase-2
GI: Gastrointestinal

## References

[1] Cavkaytar O, du Toit G, Caimmi D (2019). Characteristics of NSAID-induced hypersensitivity reactions in childhood. Pediatr. Allergy Immunol..

[2] Ghlichloo, I. & Gerriets, V. *StatPearls* (StatPearls Publishing: Copyright © 2023, StatPearls Publishing LLC., 2023).

[3] Brewer CB, Bentley JP, Hallam JS, Woodyard CD, Waddell DE (2014). Use of analgesics for exercise-associated pain: Prevalence and predictors of use in recreationally trained college-aged students. J. Strength Cond. Res..

[4] Wiliński J (2015). Non-steroidal anti-inflammatory drugs and paracetamol in self-therapy of various disorders in students of different fields of study. Folia Med. Cracov.

[5] Jarernsiripornkul N, Phueanpinit P, Pongwecharak J, Krska J (2016). Experiences of and attitudes towards receiving information about non-steroidal anti-inflammatory drugs: A cross-sectional survey of patients in Thailand. Expert Opin. Drug Saf..

[6] Babelghaith SD, Alarifi MN, Wajid S, Alhawassi TM, Alqahtani SK, Alghadeer SM (2019). Knowledge of patients on safe medication use in relation to nonsteroidal anti-inflammatory drugs. Saudi J. Anaesth..

[7] Ho KY (2020). Perceptions and beliefs regarding NSAIDs in the Asia-Pacific region. J. Pain Res..

[8] O'Connor S, McCaffrey N, Whyte E, Moran K, Lacey P (2019). Nonsteroidal anti-inflammatory drug use, knowledge, and behaviors around their use and misuse in Irish collegiate student-athletes. Phys. Sportsmed..

[9] Khatib, R. *The Most Commonly Used Drugs at the Primary Health Care Level in Palestine*. http://icph.birzeit.edu/research/publications/most-commonly-used-drugs-primary-health-care-level-palestine (accessed May 19, 2023) (2003).

[10] Khdour MR, Hallak HO, Hejaz H, Shaeen M, Dweib M (2014). Patterns of NSAIDs use in Palestinian mid-territories: A prospective study of ambulatory patients in outpatient pharmacies. Curr. Clin. Pharmacol..

[11] Qasrawi H, Assi S, Ghanim N, Zyoud SH, Al-Jabi SW (2021). A Descriptive study of pain relief practices among student-athletes in Palestine: Focus on non-steroidal anti-inflammatory drugs, and complementary medicine and alternative medicine use. J. Community Health.

[12] Arandy DA (2019). Comparative evaluation of drug information leaflets for non-steroidal anti-inflammatory drugs in Palestine: Local versus imported products. BMC Health Serv. Res..

[13] Siddig AI, Alqahtani AM, AlShalawi A, Turkistani M, Binbaz S, Altowairqi A (2020). Awareness of analgesics complications in Saudi Arabia: A cross-sectional study. Future J. Pharm. Sci..

[14] Sostres C, Gargallo CJ, Arroyo MT, Lanas A (2010). Adverse effects of non-steroidal anti-inflammatory drugs (NSAIDs, aspirin and coxibs) on upper gastrointestinal tract. Best Pract. Res. Clin. Gastroenterol..

[15] Harirforoosh S, Asghar W, Jamali F (2013). Adverse effects of nonsteroidal antiinflammatory drugs: An update of gastrointestinal, cardiovascular and renal complications. J. Pharm. Pharm. Sci..

[16] Green M, Norman KE (2016). Knowledge and use of, and attitudes toward, non-steroidal anti-inflammatory drugs (NSAIDs) in practice: A survey of ontario physiotherapists. Physiother. Can..

[17] Shaikh S, Tharani R, Saad Khan M, Chughtai MRB, Alam B (2021). Physiotherapists' knowledge, usage and attitude towards non-steroidal anti-inflammatory drugs (NSAIDs) in Karachi, Pakistan. Int. J. Risk Saf. Med..

[18] Al Masoudi W, Al Dweik R, Al-Dweik G (2023). Knowledge and practices regarding the use of non-steroidal anti-inflammatory drugs among university students in Jordan. Open Public Health J..

[19] Wawryk-Gawda E, Chylinska-Wrzos P, Lis-Sochocka M, Jodlowska-Jedrych B (2014). Consumption and awareness of students about nonsteroidal anti-inflammatory drugs. Curr. Issues Pharm. Med. Sci..

[20] Kahnamouei-aghdam F, Farzaneh E, Skandaroghli B, Fathi A (2015). Nonsteroidal Anti-inflammatory drugs (NSAIDs) use among university students in Ardabil City, 2014. J. Behav. Health.

[21] Farah RI (2023). Pattern of use and awareness of side-effects of non-steroidal anti-inflammatory drugs in the Jordanian population. Ann. Med..

[22] Sulaiman W, Seung OP, Ismail R (2012). Patient's knowledge and perception towards the use of non-steroidal anti-inflammatory drugs in rheumatology clinic Northern Malaysia. Oman Med. J..

[23] An-Najah National University. *Facts and Figures. 2023*. https://www.najah.edu/en/about/annu-facts/ (accessed May 19, 2023).

