# Supplementary material for: Prevalence, awareness, and patterns of non-steroidal anti-inflammatory drug use among health science students in Palestine: a cross-sectional study
Source: Sci Rep. 2023 Nov 13;13:19844. doi: 10.1038/s41598-023-47279-2 (PMC10646118; doi:10.1038/s41598-023-47279-2)
Supplement: Supplementary file 1 — Supplementary Information. [file 41598_2023_47279_MOESM1_ESM.docx]

**Additional File 1: Study questionnaires.** This is the final version of the English version that was used to assess the prevalence, awareness, and patterns of non-steroidal anti-inflammatory drug use among college of health science students in Palestine.

**English version**

Dear participant,

The purpose of this questionnaire is to identify and shed light on the level of awareness and consumption of NSAIDs by medical college students. Additionally, we aim to identify the factors influencing this consumption, strategies to address them, and the extent of their impact on students' lives. We kindly request that you answer the following questions.

Please note that the information you provide will remain confidential and will be used solely for scientific research purposes. We appreciate your cooperation in advance.

Note: The form is written in the masculine form, but it is directed to both sexes equally.

| **Commercial medicines containing NSAIDs** | | | | |
| --- | --- | --- | --- | --- |
| - Trufen | - Neurofen | - Diclofen | - Mobicol | - Etoricoxib |
| - Trufen plus | - Naprex | - Voryn | - joflam | - Arcoxia |
| - Neurofen plus | Dolocare | - Voltaren | - Cataflam | - Tericox |
| - Neurofen cold and flu | - Pirox | - Rufenal | - Toleran | - Etoflam |
| - Extrafen | - Exipan | - Abitren | - Anaflam | - Nabuco |
| - Ultrafen plus | - Feldene | - Swiss relief | - Celecoxib | - Relifex.. |
| - Ultrafen | - Prexin | - Arthrotec (with misoprostol) | - Coxib | - Etodolac |
| - Isofen | - Pirox | - Ketorolac tromethamine | - Celex | - Naxyn |
| - Ibusama | - Moxicam | - xefo | - Celebra | - Naproxan |
| - Ibufen | - Movalis | - Oxidur | - Celecox | - Narocin |
| - Etodolac | - Mesulid | - Profenid | - Celecoxib | - Point |
| - Advil | - Indolin | - Indocaps | - Indivis | - Indopharm |

This is a list of the names of the most prominent commercial medicines available in Palestinian pharmacies, which contain in their composition NSAIDs or Paracetamol

Dear participant, please put a tick next to the name of the non-steroidal analgesic that you are using

| **Commercial medicines containing paracetamol** | |
| --- | --- |
| - Otamol | - paramol |
| - Tailol | - Paramol extbra |
| - Novimol | - Paramol plus |
| - Acamoli | - Revamol |
| - Acamol | - Dexamol |
| - Panadol | - Exidol |
| - Febramol | - Extrafen |
| - Abrol | - Migrane |
| - Sedamol | - dexamol cold |
| - Sensamol | - Dexamol plus |
| - Paracod | - Tylenol |

Dear participant, you can put a check next to more than one option for questions that have more than one answer, and if you do not find your answer within the options, you can write it next to the question

**This form consists of eight sections:**

**Section One:**

|  |  |  | **......................** | 1. **Age** |
| --- | --- | --- | --- | --- |
|  | **Female** |  | **Male** | 1. **Gender** |
|  | **.............** | 1. **Weight** | **........................** | 1. **Hight** |
|  | **City** | **Village** | **Camp** | 1. **Residency** |
|  | **Others**  **(…….)** | **With my family** | **Alone** | 1. **Living status** |
| **Widower** | **divorced** | **married** | **single** | 1. **Social status** |
| **Nursing** | **Laboratory science** | **Pharmacy** | **Medicine** | 1. **Specialization** |
| **Speech and hearing** | **Optics** | **Physiotherapy** | **Doctor of pharmacy** | **Midwifery** |
|  | **Governmental insurance** | **Private insurance** | **I don’t have insurance** | 1. **Health insurance** |
| **More than 10000** | **5000-10000NIS** | **2000-5000 NIS** | **Less than 2000 NIS** | 1. **Income** |
| **Forth year** | **Third year** | **Second year** | **First year** | 1. **Education level** |
|  |  | **Sixth year** | **Fifth year** |  |

**Second section:**

| \|  \| **No** \|  \| **Yes** \| 1. **Have you ever used NSAIDs?** \| \| \| \| --- \| --- \| --- \| --- \| --- \| --- \| --- \| \|  \|  \| 1. **If the answer is yes, how often do you take NSAIDs?** \| \| \| \| \| \| **Several times a week** \| **Once a week** \| **Several times a month** \| **Once a month** \| \| **Several times ayear** \| **Once a year** \| \| | | | | | | | | | | | | | | | | | | | | | |  |  |
| --- | --- | --- | --- | --- | --- | --- | --- | --- | --- | --- | --- | --- | --- | --- | --- | --- | --- | --- | --- | --- | --- | --- | --- | --- | --- | --- | --- | --- | --- | --- | --- | --- | --- | --- | --- | --- | --- | --- | --- | --- | --- | --- | --- | --- | --- |
|  | |  | | |  | |  | | | | |  | | | |  |  |  |  |  |  |  |  |
| **Third section :**  **What are the cases that may require using NSAIDs or paracetamol or both** | | | | | | | | | | | | | | | | | | | | | | |  |
|  | | |  | | | | | |  | |  |  |  |  |  |  |  |  |  |  |  |  |  |
| **Using paracetamol** | | | | | | **Using NSAIDs** | | | | | | | | **Case** | | | | | | |  |  |  |
|  | | | | | |  | | | | | | | | **Cold cases** | | | | | | |  |  |  |
|  | | | | | |  | | | | | | | | **Toothache** | | | | | | |  |  |  |
|  | | | | | |  | | | | | | | | **Fever** | | | | | | |  |  |  |
|  | | | | | |  | | | | | | | | **Stomach pain or heartburn** | | | | | | |  |  |  |
|  | | | | | |  | | | | | | | | **Muscle pain** | | | | | | |  |  |  |
|  | | | | | |  | | | | | | | | **Stress** | | | | | | |  |  |  |
|  | | | | | |  | | | | | | | | **Headache** | | | | | | |  |  |  |
|  | | | | | |  | | | | | | | | **Asthma** | | | | | | |  |  |  |
|  | | | | | |  | | | | | | | | **Menstrual pain** | | | | | | |  |  |  |
|  | | | | | |  | | | | | | | | **Post traumatic pain (bruises, ankle sprain)** | | | | | | |  |  |  |
|  | | | | | |  | | | | | | | | **Backache** | | | | | | |  |  |  |
|  | | | | | |  | | | | | | | | **Irritable bowel syndrome** | | | | | | |  |  |  |
|  | | | | | |  | | | | | | | | **Skin rashes** | | | | | | |  |  |  |
| **What are the pharmaceutical forms that you used to use from NSAIDs?** | | | | | | | | | | | | | | | | | | | | | | | |
|  | - **Effervescent tablet** | | | | | | | - **injections** | | | | | | | - **Pills** | | | | |  |  | | |
| **Where do you get NSAIDs from?** | | | | | | | | | | | | | | | | | | | | | | | |
| - **friends** | | | | - **University clinic** | | | | | | - **Doctors** | | | | | | | | - **pharmacy** | | | | | |
|  | | | |  | | | | | | - **Leftover previous medications** | | | | | | | | - **Family medications** | | | | | |
|  | | | | **Do you take NSAIDs in conjunction with any of the following substances?** | | | | | | | | | | | | | | | | | | | |
| - **Other medications** | | | | - **Coffee** | | | | | | - **Herbs** | | | | | | | | - **Alone** | | | | | |
|  | | | |  | | | | | |  | | |  | | | | |  |  | | | | |

**Fourth section:**

| **Do you think it is generally safe to use NSAIDs?** | |
| --- | --- |
| - **No** | - **Yes** |
| **Do you know the side effects of using NSAIDs?** | |
| - **No** | - **Yes** |
| **Do you think NSAIDs can be purchased without a prescription?** | |
| - **No** | - **Yes** |
| **Do you think that easy access to NSAIDs ‘as happens these days’ is suitable?** | |
| - **No** | - **Yes** |
| **Do you think it is appropriate to buy NSAIDs from different places such as the supermarket?** | |
| - **No** | - **Yes** |
| **Do you think NSAIDs are being abused at present?** | |
| - **No** | - **Yes** |
| **Do you think it is appropriate to market anti-inflammatory drugs via social media?** | |
| - **No** | - **Yes** |

**Fifth section:**

|  | | **When using NSAIDs, do you use them continuously or intermittently?** | | | |
| --- | --- | --- | --- | --- | --- |
|  | |  | - **Intermittently** | | - **Continuously** |
| **How to use painkillers?** | | | | | |
|  | |  | - **Intermittently** | | - **Continuously** |
| **If the pain does not go away, what do you do ?** | | | | | |
| - **take another painkiller** | - **take a second dose** | | | - **ask a pharmacist** | - **Ask a doctor** |
| **When you buy NSAIDs without a prescription, what is your first source of information about the drug?** | | | | | |
| - **Family** | - **Media** | | | - **Books and research** | - **Doctors or pharmacist** |
|  |  | | | - **Medication leaflets** | - **Friends** |
| **Do you think that increasing the dose of painkillers has side effects?** | | | | | |
| - **I don’t know** | | | | - **No** | - **Yes** |

**Sixth section:**

| **Is the incorrect use of NSAIDs may harm the kidneys** | | |
| --- | --- | --- |
| - - - - **I don’t know** | - - - - **No** | - **Yes** |
| **Is the incorrect use of NSAIDs harmful to blood pressure and the heart?** | | |
| - **I don’t know** | - **No** | - **Yes** |
| **Is the incorrect use of NSAIDs may cause an acceleration in the growth of cancer cell?** | | |
| - **I don’t know** | - **No** | - **Yes** |
| **Is the incorrect use of NSAIDs may cause stomach ulcers?** | | |
| - **I don’t know** | - **No** | - **Yes** |
| **Is the incorrect use of NSAIDs may cause bleeding in the gastrointestinal tract?** | | |
| - **I don’t know** | - **No** | - **Yes** |
| **Is the incorrect use of NSAIDs may harm the respiratory system?** | | |
| - **I don’t know** | - **No** | - **Yes** |
| **Is the use of NSAIDs safe in all stages of pregnancy?** | | |
| - **I don’t know** | - **No** | - **Yes** |

**Seventh section:**

**Do the side effects of taking NSAIDs increase in the elderly?**

**• I don’t know • No • Yes**

**Does taking a high dose of NSAIDs increase their side effects?**

**• I don’t know • No • Yes**

**Does taking NSAIDs continuously increase their side effects?**

**• I don’t know • No • Yes**

**Does taking more than one type of NSAID at the same time increase its side effects?**

**• I don’t know • No • Yes**

**Are the side effects of taking NSAIDs increased in patients with high blood pressure and heart disease?**

**• I don’t know • No • Yes**

**Are smokers more likely to experience side effects from the use of NSAIDs?**

**• I don’t know • No • Yes**

**Eighth section:**

**Choose your Attitudes toward receiving information about the side effects of NSAIDs**

| **Strongly disagree** | **disagree** | **Iam not sure** | **Strongly agree** | **Agree** | **Question** |
| --- | --- | --- | --- | --- | --- |
|  |  |  |  |  | **It is important to have an idea about medication side effects** |
|  |  |  |  |  | **Informing you of the side effects of the medication may increase your level of anxiety** |
|  |  |  |  |  | **The doctor should have a role in informing you about the side effects of the medicines** |
|  |  |  |  |  | **The pharmacist has a direct role to play in providing information about the side effects of the medicine** |
|  |  |  |  |  | **Having information about the side effects of medication helps the patient to inform the doctor about the abnormal symptoms that may appear** |
|  |  |  |  |  | **Having information about the side effects of your medicine may cause you to stop using the medicine** |
|  |  |  |  |  | **Receiving information from the drug leaflet may encourage noncompliance with the medicine** |
|  |  |  |  |  | **The drug leaflet is a source of information through which it is possible to monitor the side effects of a medicine and inform the doctor about them** |
|  |  |  |  |  | **Receiving information from the drug leaflet if a medicine is used for the first time is necessary** |
|  |  |  |  |  | **The leaflet information is an inaccurate source of medication information for you** |
